# Positioning of Weight Bias: Moving towards Social Justice

**DOI:** 10.1155/2016/3753650

**Published:** 2016-09-22

**Authors:** Sarah Nutter, Shelly Russell-Mayhew, Angela S. Alberga, Nancy Arthur, Anusha Kassan, Darren E. Lund, Monica Sesma-Vazquez, Emily Williams

**Affiliations:** ^1^Counselling Psychology, Werklund School of Education, University of Calgary, Calgary, AB, Canada; ^2^Werklund School of Education, University of Calgary, Calgary, AB, Canada; ^3^Curriculum and Learning, Werklund School of Education, University of Calgary, Calgary, AB, Canada

## Abstract

Weight bias is a form of stigma with detrimental effects on the health and wellness of individuals with large bodies. Researchers from various disciplines have recognized weight bias as an important topic for public health and for professional practice. To date, researchers from various areas have approached weight bias from independent perspectives and from differing theoretical orientations. In this paper, we examined the similarities and differences between three perspectives (i.e., weight-centric, non-weight-centric (health-centric), and health at every size) used to understand weight bias and approach weight bias research with regard to (a) language about people with large bodies, (b) theoretical position, (c) identified consequences of weight bias, and (d) identified influences on weight-based social inequity. We suggest that, despite differences, each perspective acknowledges the negative influences that position weight as being within individual control and the negative consequences of weight bias. We call for recognition and discussion of weight bias as a social justice issue in order to change the discourse and professional practices extended towards individuals with large bodies. We advocate for an emphasis on social justice as a uniting framework for interdisciplinary research on weight bias.

## 1. Introduction

Weight bias, or the stigma towards and negative stereotypes about individuals who have large bodies, has been described as “overt, expressible, and widely held” [1, p. 891]. Researchers have found weight bias to be the fourth most frequently reported form of discrimination and determined a 66% increase in its occurrence between 1995 and 2006 [2]. Individuals who live in large bodies are stereotypically characterized as being lazy, sloppy, weak-willed, physically and sexually unattractive, and gluttonous [3]. Research has documented weight bias in the attitudes of health care professionals [4], success in education [5], hiring practices in the workforce [6], interpersonal relationships of individuals with large bodies [7], and the influence of the media on weight bias [8]. Research has also documented numerous physical and psychological health consequences of weight bias, including, but not limited to, increased stress [9], decreased motivation to engage in physical activity [10], increased binge-eating behaviour [11], and depression [12].

Research areas such as fat studies, health care, and psychology have recognized weight bias as an important issue for research and professional practice [13]. However, despite the common interest in weight bias, each area remains relatively segregated to the point where each perspective may be associated with specific journals as the common outlets for research and review articles, with little overlap. Activists and researchers have been described as being “fundamentally engaged in framing contests over the nature and consequences of excess body weight” [14, p. 869]. Saguy and Riley [14] posited that, when such framing contests represent opposing positions, it may undercut the integration of the insights achieved by the conflicting perspectives. Consequently, few attempts have been made to name common interests for moving forward with interdisciplinary research.

Previous researchers have noted that body weight is often framed in one of two opposing ways: through the lens of body diversity or through the lens of excess weight as a preventable health risk [14, 15]. Each way of framing body weight constructs a different social problem and entails a different solution [14]. Each of the various perspectives engaged in weight bias research takes a unique position in understanding and explaining the origins and consequences of weight bias. Further, research areas also have different approaches to language, with some more frequently using the word* fat* to refer to individuals who have large bodies and others more frequently using the words* overweight* or* obese*. Differences are also present with respect to preference for person-first (e.g.,* person with obesity*) or identity-first (e.g.,* fat person*) language. In recent years, person-first language has become increasingly popular, as researchers have proposed that person-first language promotes openness and aids in the reduction of stigma and prejudice [16, 17]. This discussion has continued, however, as scholars in the area of disability studies have also argued for the continued use of identity-first language together with person-first language as a way to claim identity and to promote pride [17]. Proponents of using both person-first and identity-first terms flexibly have stated that it may acknowledge the roles and perspectives of different groups and that it will also allow for the opportunity to ask about preferred terms [17]. In relation to the field of weight bias, this may resemble openness to using* both* person-first (e.g.,* person with obesity*) and identity-first (e.g.,* fat person*) language, depending on the context as well as individual preference.

The purpose of this paper was to conduct a literature review of selected disciplines that contribute to weight bias research in order to overview the common, potentially competing, and emerging perspectives on this topic. Weight bias research was selected from multiple research disciplines, including fat studies, feminist literature, health at every size (HAES), health care, psychology, and sociology. The review targeted papers that provided a current conceptual and theoretical overview of different perspectives of weight bias. The similarities among, and differences between, each of these perspectives were examined with regard to five research questions: (a) what is the language used to refer to weight and individuals with large bodies? (b) through what general lens is discussion of weight bias approached? (c) what theoretical positions are used to discuss the source or causes of weight bias? (d) what consequences of weight bias are identified? and (e) how are these consequences discussed with regard to influence on weight-based inequity? In the following discussion, we introduce three perspectives of weight bias with regard to these five questions: the weight-centric perspective, the non-weight-centric perspective, and health at every size perspective. In addition to these perspectives, we also provide a brief overview of literature pertaining to the social determinants of health, as it supports our discussion of weight-based inequity identified among the three perspectives. Finally, we introduce a focus on social justice as a uniting perspective that offers a foundation for advancing interdisciplinary research on weight bias.

## 2. Differing Perspectives of Weight Bias

Saguy and Riley [14] described disagreements that surface when obesity is framed from different perspectives. For example, groups that understand obesity through a body diversity framework, versus a preventable risk framework, would have varying opinions about “if or why higher weights have adverse health consequences, what an ideal weight is or whether a universal ideal weight even exists, why people gain weight, why some weigh more than others, and whether weight loss improves health” (p. 874).

These body diversity and preventable risk frameworks have been commonly identified in the literature using the terms* weight-centric* and* non-weight-centric or health-centric* [18–20]. Researchers have also referred to these frameworks as* weight-normative* and* weight-inclusive* [21]. For the purposes of this paper, these terms will also be used to frame our discussion, with clarification. The term weight-centric has been previously defined as having the six following tenants: (a) the belief that weight is under individual control, (b) the belief that weight gain is caused by an imbalance in caloric intake and energy usage, (c) the belief that health status can be predicted by weight, (d) the belief that excess body weight causes disease and early death, (e) the belief that methods for successful long-term weight loss involve the modification of eating and exercise patterns, and (f) the belief that losing weight will result in better health [20]. However, for the purposes of this paper, the term weight-centric will be used to refer to research that typically discusses weight bias through a consideration of body weight and increasing rates of overweight and obesity, without the assumption of the six tenants described by O'Hara and Gregg [20]. Finally, it is also important to note that the perspectives described below are not mutually exclusive but may be regarded as emerging distinctions within the field of weight bias.

The literature represented in this paper provides a brief overview of each perspective and is not intended to be a comprehensive literature review. Articles for this paper were identified through two large multidisciplinary databases (i.e., Academic Search Complete and Web of Science), with a focus on identifying articles that presented a broad theoretical overview of one or more perspectives in addition to more focused experimental and correlational study-based articles. Multiple keyword search terms were used to aid in the collection of a broad range of articles representing each perspective. Various combinations of the following keyword search terms were used: anti-fat bias, body diversity, body size, body weight, epidemiology, fat acceptance, fat bias, fat oppression, fat shaming, fat stigma, fat studies feminism, health at every size, inequality, obesity, obesity stigma, weight bias, and weightism. This resulted in 173 articles that were reviewed for examination and comparison of the differing perspectives of weight bias that informed the current discussion. Articles were then scanned for the language used to refer to weight and individuals with large bodies as well as the theoretical orientation of the article in order to determine the perspective that the article best represented.

### 2.1. The “Weight-Centric” Perspective

The weight-centric perspective is characterized by the investigation of weight bias through the lens of increased prejudice and discrimination based on body weight, influenced in part by increasing rates of obesity [4, 22]. This perspective often includes researchers from fields such as health care and psychology, who identify weight bias as a prevalent social issue in need of further understanding [23]. Much of the research from this perspective has focused on documenting the prevalence of weight bias and discrimination within life domains (e.g., employment) [6] and among health care professionals and preprofessionals [4, 24]. Researchers have also examined the association of weight bias with other attitudes, such as racism and physical appearance concerns, as well as fundamental beliefs that serve as the basis for a political, economic, or social system [1, 25].

When referring to weight, researchers from the weight-centric perspective tend to use the terms* overweight* and* obesity*. When referring to individuals with large bodies, researchers tend to use both person-first (i.e.,* person with obesity*) [26, 27] and identity-first (i.e.,* obese person*) [8, 28] language. Professional associations such as the American Psychological Association encourage the use of person-first language [16] and person-first language has become increasingly popular within the medical community [29].

It is important to note that the current weight-centric perspective on weight bias has grown from a strong history of research documenting weight bias, especially among health professionals [4] and that this perspective has been, and continues to be, critiqued due to the “medicalization” of body weight. Weight has been used as an easily measurable proxy for health and, as such, individuals' weight and health have often been confounded. More specifically, researchers have critiqued obesity research as contributing to the belief that weight is within individual control, that it is as simple as calories in versus calories out, that weight and health are inextricably related, that excess weight causes disease and death and losing weight will result in health, and that methods for long-term weight loss are known and effective [15, 20, 30, 31].

#### 2.1.1. Attribution Theory

Although there is no consensus within this perspective, researchers tend to utilize attribution theory to understand the origins of weight bias [4]. Attribution theory proposes that individuals are motivated to seek out causal explanations for outcomes or conditions and that negative bias towards individuals with large bodies arises from the tendency for weight to be regarded as under personal control [1, 22]. Research in attribution theory has suggested that when conditions are regarded as uncontrollable, individuals are likely to display increased liking and pity but that when conditions, such as obesity, are perceived as controllable, individuals are likely to display dislike, anger, and negative judgments [32]. Such reactions, specifically feelings of disgust, have been linked to weight bias and the treatment of individuals with large bodies [33]. Many researchers have regarded weight bias as occurring due to the view of obesity as a behavioural disorder that ignores the complexity of its causation and positions it as something to be treated or eradicated through behavioural change [3, 27, 34]. Researchers have asserted that complex biological processes are often overlooked in favour of common misconceptions, such as obesity being the result of low physical activity or an unhealthy diet and that anyone with willpower can lose weight [34]. Further, the success of behaviour modification obesity interventions is often measured through how much weight a person loses [35]; however, weight is not a behaviour. It would seem that the focus on the outcome of weight loss is measured without the consideration of the complex biological and contextual influences and that individual willpower and behaviour are commonly regarded as the cause of obesity. Research documenting the media effects of such “controllability” messages [8] as well as experimental investigations [36] has provided support for attribution theory.

#### 2.1.2. Thin-Ideal Internalization

More recently, researchers from the weight-centric perspective have also proposed that weight bias reflects the degree to which an individual has bought into social standards of attractiveness [10]. Over the last several decades, ideal body standards have shifted, with the ideal body for women being prescribed as thin, with large breasts and toned muscles, and the ideal body for men being prescribed as lean and muscular, with wide shoulders and a narrow waist [37–39]. Recent research has provided support for the relationship between the internalization of thin-ideal body standards and weight bias [40]. For example, the physical appearance concerns related to the internalization of the thin-ideal may serve to increase disgust reactions towards individuals with large bodies, thus increasing weight bias [41].

#### 2.1.3. Social Comparison Theory

Researchers have also suggested that weight bias is influenced by the tendency to make comparisons based on body weight [42]. Social comparison theory asserts that individuals are motivated to compare and evaluate their own opinions and abilities with the opinions and abilities of others, which instills a sense of validation [43]. Previous researchers have suggested that weight bias is a form of downward social comparison that serves to increase the self-esteem of the individual making the comparison [42]. For example, a social comparison perspective of weight bias asserts that individuals may compare their bodies to people who have larger bodies in order to feel better about their own body size, thus perpetuating weight bias.

#### 2.1.4. Consequences of Weight Bias

Researchers operating from the weight-centric perspective have also identified significant negative consequences of weight bias. Among these, researchers have identified depression [12], increased binge-eating behaviour [11], anxiety [44], and stress [9], as well as decreased motivation to engage in physical activity [10]. Although researchers from this perspective have focused less on how weight bias influences social inequity, researchers have suggested that negative beliefs and judgments tend to be greater for girls and women with large bodies compared to men [45, 46]. Research has also suggested that weight bias can impact patient quality of care as well as equality of patient care, as health care professionals may spend less time with patients with large bodies and provide fewer treatment options [47]. Finally, it is important to note that researchers have stated that the consequences of experiencing weight bias may be more harmful than simply having a large body and that perhaps the health consequences attributed to obesity may be better explained by weight bias [48].

### 2.2. The “Non-Weight-Centric” or “Health-Centric” Perspective

The non-weight-centric perspective is characterized by the tendency to approach discussion of weight and weight bias by taking a critical stance to the popular obesity discourses [49, 50]. This perspective often includes researchers from interdisciplinary fields such as fat studies and feminist studies, as well as sociology, who identify that weight has become an indicator for social status whereby large bodies are regarded as less successful and less attractive [30]. Researchers from this perspective seek to understand discourses beyond the medicalization of obesity and to build a body-accepting and embodied culture [30, 49, 50], where the focus is on health, not weight. In other words, from this perspective, body size is conceptualized as existing on a continuum of natural body diversity and is not something that is to be corrected.

Researchers from the health-centric perspective examine the broader social forces that aid in shifting attention and concentration “away from the fat body itself and more towards positioning and contingent systems and structures” [49, p. 1020-1021]. More specifically, researchers in this area have examined weight bias in relation to a broad array of topics including body shame and sexual health [51, 52], media influence [53–55], the fashion industry [56], education [57–59], public health [60, 61], and health care [62]. In addition, feminist researchers have also been critical of the lack of attention paid to weight bias in relation to the attention paid to other weight-related topics, such as eating disorders [63]. Vincent Roehling [64] proposed that weight bias may be an issue more congruent with the second wave of feminism, in that it tends to impact White middle-class women the most, and may be incompatible with mainstream feminist thought that has emphasized the influences of gender, race, socioeconomic status, sexual orientation, and their intersections. Shaw and Lee [65] described current feminist thought as being inclusive and affirming of all women, as well as seeking to promote equality and justice for all women. Not only is an increased focus on weight within feminist thought important with regard to weight bias, but also it might highlight the similar barriers and systemic issues that create the social conditions for the occurrence of both eating disorders* and* weight bias.

When referring to weight, researchers from the health-centric perspective tend to use terms such as* fat*,* fatness*, or* fat bodies* as well as identity-first (i.e.,* fat person*) [15, 30] language when referring to individuals with large bodies. Many researchers and activists from this perspective use the word* fat* intentionally as a way to reclaim power, to remove shame and stigma, and to position fat as one of many possible identity descriptors [15, 49]. In addition to being critical of the language used to describe weight and individuals with large bodies, researchers from the fat perspective examine weight bias through numerous theoretical approaches, including critical analysis of obesity discourses [30], critical fat studies [15, 66], feminist poststructuralism [62], and intersectionality [67]. Each of these theoretical approaches will be discussed below.

#### 2.2.1. Critical Analysis of Obesity Discourse

Researchers who have approached weight bias through a critical examination of obesity discourses have identified two competing frames. The first frame is considered the dominant weight and beauty discourse, which recognizes the “slender” body as attractive and successful and large bodies as unhealthy, diseased, and representing failure, which results in body size being regarded as an individual responsibility [15, 30, 31]. Alternatively, the second frame recognizes this discourse as originating in Western patriarchal culture and that these widespread messages serve to devalue female body size and shape with serious consequences [30, 31]. Despite these two competing discourses, researchers have recognized that “obesity epidemic” discourse tends to dominate public discussion [30, 31]. This discourse is problematic, as the ongoing discussion of large bodies as an “epidemic,” “infection,” or a “plague” leaves no room for the acceptance of natural body diversity. By being complicit with this discourse of the “obesity epidemic,” researchers have asserted that large bodies will continue to be excluded, marginalized, and regarded as immoral [15, 31].

#### 2.2.2. Critical Fat Studies

Similar to the critical analysis of obesity discourses, critical fat studies have been described as a position that disputes weight bias in two ways [61]. First, by placing emphasis on the complex nature of body weight, critical fat studies challenges the dominant framing of obesity as an unbalanced relationship between calories in and calories out and as a personal choice [44, 61]. Rather, critical fat studies reframes body weight as a natural form of body diversity [14]. Second, critical fat studies seeks to challenge the assumption that large bodies are a serious threat to individual health as well as other “universal scientific ‘truths'” often presented in research consistent with the dominant obesity discourse [15, p. 100, 66]. More specifically, researchers from this perspective challenge incorrect and biased assumptions about weight and body size that occur in research, policy, entertainment media, news media, and public health campaigns [15].

#### 2.2.3. Feminist Poststructuralism

Many researchers within the non-weight-centric/health-centric perspective who operate within feminist studies approach the discussion of weight bias using poststructuralism, which emphasizes the examination of how the body and identity are shaped through social relations as well as cultural and institutional beliefs [30, 62]. This framework allows for the consideration of diverse perspectives like race, ethnicity, socioeconomic status, and ability, among others, and “provides a lens for an in-depth examination of personal experiences, relationships and contextual meanings of relations of power between individuals” [62, p. 1188].

#### 2.2.4. Intersectionality and Influences on Social Inequity

Finally, many researchers also conduct research through the lens of intersectionality, which has been defined as the examination of the interactions of multiple forms of oppression and the outcome of these interactions with regard to power [50, 67, 68]. This research has allowed for further understanding of the differential effects that weight bias has on social equality with regard to gender, race, socioeconomic status, and sexual orientation [66, 67]. Many researchers have maintained that weight bias is “deeply gendered” and “that body size matters* differently* for men and women in the identity positions available to them” [31, p. 159]. Nurka [55] asserted that a slender body is one of the demands of “good womanhood” and that it is connected with feminine identity and success (p. 168). Weight bias and its intersections have been described by researchers from the non-weight-centric perspective as contributing to an oppressive social context for individuals with large bodies, where large bodies are visual representations for immorality [49, 69].

In addition to acknowledging that weight bias contributes to an oppressive social context, researchers have identified other consequences of dominant weight discourses for women with large bodies, including weight-based harassment and social exclusion [30], disordered relationships with food [70], the draining of women's energy and resources with regard to seeking weight loss and fad diets [58], normalizing female body anxiety [55], and the reinforcement of the cultural belief that women should control their desires [71].

### 2.3. Health at Every Size

Health at every size (HAES) has been described as a non-weight-centric, transdisciplinary movement [18, 19] comprising researchers from all disciplinary backgrounds, who approach weight bias through the consideration of weight and the position that societal obsessions with thinness and dieting are unhealthy and do not allow for natural body diversity [72]. Rather than focusing on weight loss, the HAES paradigm focuses on health promotion and improving the emotional, physical, and spiritual well-being of individuals of all sizes [18, 58]. Specifically, researchers from this area promote self-acceptance, body diversity, and improved health behaviours regardless of size, such as engaging in physical activity for pleasure and intuitive eating behaviours [18, 58]. HAES researchers have critically examined and challenged weight-related assumptions and the use of body mass index (BMI) as an indicator of health and have provided evidence for the need for a paradigm shift within health care [18, 73]. More specifically, HAES interventions do not focus on weight as an outcome; rather, behavioural health indices such as intuitive eating, body esteem, and psychological functioning are more relevant measures of success [74, 75].

Although the HAES paradigm is relatively new, with the majority of research published beginning in the year 2000 [76], researchers from this perspective have commented on the potential clinical application of the HAES paradigm [72, 73], the application of HAES to public health [19, 76] and dietetics [77], and have examined the long-term effects of HAES interventions [78]. When conducting research within the paradigm, HAES researchers tend to use language consistent with both perspectives when referring to both weight (e.g.,* overweight*,* obesity*, and* fat*) and individuals with large bodies (e.g.,* obese person* and* fat person*) [18, 19, 79], as well as the term* people of size* [80].

#### 2.3.1. “Healthism”

HAES researchers have approached the understanding of weight bias through a critical examination of “healthism.” Researchers have stated that healthism has cemented discourses of health and weight as being under the almost exclusive control of the individual, who is viewed as having the responsibility and obligation to strive for the perfect body [79, 81]. This healthism, and the accompanying fitness movement, prescribes specific practices and beliefs that serve to “externally regulate individual and collective bodies as well as reinforce the internalization of bodily self-control in the name of health” [79, p. 358]. Further, researchers have asserted that the dominant healthism discourse and the strive for bodily perfection intentionally exclude the consideration of the social determinants of health, such as socioeconomic status, employment status, education, lack of access to physical activity, and lack of access to health care [79, 82].

#### 2.3.2. Consequences of Weight Bias

HAES researchers have maintained that the discourses of health and weight that contribute to weight bias have significant consequences for individuals with large bodies, including being regarded as unattractive and unhealthy [79], demotivating healthy behaviours [18], and avoidance of health care [83]. Through this perspective, weight bias is regarded as, in part, responsible for “reinforcing and privileging slimness in a culture that promotes health at one size” [18, p. 358]. Further, healthism and weight bias also serve to decontextualize health from the structural and social forces that impact people's lives, with serious consequences for the health care and health outcomes of individuals with large bodies [79, 82].

### 2.4. Summary

The above review of the three emerging perspectives and approaches in weight bias research suggested that each perspective approaches discussion of weight bias from a unique point of view. However, similarities among these perspectives exist with regard to how weight bias is conceptualized and understood. In addition there are similarities and differences between the perspectives with regard to how weight bias is termed, the consequences of weight bias, and how these consequences influence social inequity.  Table 1 presents a summary of this discussion.

## 3. Social Determinants of Weight: A Fourth Perspective for Consideration?

In the early 2000s, research in public health and epidemiology began to rapidly multiply [84], with researchers consistently documenting the social and environmental disparities in health [85]. Diez Roux and Mair [85] discussed the trends driving the increased interest in neighbourhoods and health. First, they posited that researchers have increasingly recognized that explanations focusing solely on the responsibility of the individual for ill-health are inadequate and fail to capture the complex social and structural factors that contribute to weight. Second, Diez Roux and Mair [85] suggested that researchers have experienced a renewed interest in examining the causes of social inequities in health, including but not limited to differences between racial and ethnic groups. Third, researchers and policy makers have increasingly begun to consider the health effects of policies such as housing or urban planning policies [85].

Results from research investigating the social and environmental disparities in weight have suggested that factors including socioeconomic status, the neighbourhood environment, race and ethnicity, gender, and level of education all influence disparities in weight [86, 87]. Researchers from various countries around the world have observed that the rates of obesity are highest among individuals from nondominant racial and/or ethnic backgrounds as well as individuals of low socioeconomic status [87, 88]. In addition to these factors, researchers have also suggested that having a low level of educational attainment and being female also play a significant role in the social patterning of weight [89, 90]. Finally, these researchers have also examined the role of the neighbourhood environment with regard to weight disparity and have suggested that neighbourhoods with lower density of sports facilities and supermarkets, lower availability of fair-priced healthy food, and decreased walkability, as well as greater perceived hazards, show an increase in the incidence of obesity [84, 86]. Congruent with the lens of intersectionality, the highest rates of obesity occur among the most disadvantaged individuals—those who experience disadvantage in two or more areas of inequity [88].

Although the majority of epidemiology researchers documenting the disparities with regard to weight have focused discussion on overweight and obesity specifically, some researchers have also discussed the impact of these social disparities with regard to weight bias [91]. In their literature review on the relationship between gender, socioeconomic status, and obesity, Broom and Warin [92] suggested that women have the most to lose from social and economic disparities in weight, especially with regard to weight bias in employment. Faeh et al. [93] conducted a longitudinal study of education, income, and occupational class on obesity and suggested that, together with disparities in weight, the increased “mediatization” of the thin-ideal could strengthen weight bias and discrimination in the workplace, especially for women. Given the social and psychological health consequences associated with weight bias, researchers have concluded that the increasing disparities in weight are a critical public health problem, adding cumulative disadvantage to vulnerable individuals [91, 94]. As such, we would argue that one of the fundamental concepts that could bind competing perspectives on weight bias is an examination of social justice.

## 4. Weight Bias as a Social Justice Issue

Although the research areas reviewed above may differ with regard to the theoretical lens used to understand weight bias and the language used and identify different consequences and social inequities resulting from weight bias, each research area has recognized weight bias as an important social issue. Despite this recognition, however, weight bias has yet to be widely discussed as an important* social justice* issue.

Although the term social justice is not often used when discussing weight bias, a strong history of research from each perspective described above has situated it as such. Researchers have documented the many negative assumptions and stereotypes based on body weight [3] have demonstrated that weight bias has increased in recent decades [2] and have consistently established the systematic occurrence of weight bias within society, such as within the media, health care, education, employment, and interpersonal relationships [3]. Researchers have also pointed to the broad social forces that continue to reinforce the power and privilege given to thinness, which serve to deny natural body diversity [14, 30, 62, 67, 79]. Results have also documented the significant physical and mental health consequences of being victims of weight bias and discrimination [3, 79]. Finally, researchers have established the influence of weight bias on social inequity, with differences in disparity influenced by gender, race, socioeconomic status, and sexual orientation [66, 67], which may especially influence access to, and quality of, health care [47]. In our collective effort towards weight-based equality, it may seem as though* social justice*, a term used to describe the value emphasizing equitable opportunity, action to amend systemic oppression, and participation of all individuals in order to aid them in achieving maximum potential [95, 96], has been missing from our research.

In recent years, few researchers have recognized weight bias as an important social justice issue to be addressed in research, policy, and practice [13, 49, 69, 97]. In discussing weight bias as a social justice issue, van Amsterdam [31] recognized that differences in ability, race, and gender have previously been framed as solely biological in nature to justify systemic oppression and that social justice activism has resulted in the recognition of these categorizations as socially constructed.

Previous researchers have stated that weight bias is “socially sanctioned bigotry” [70, p. 250] and that “if we care about social justice, we need to figure out whether the suffering of fat people in virtue of violating the thinness norm is permissible” [50, p. 221]. Young [70] argued that the greater social system and the “perpetrators” of weight bias are unable to adequately and effectively discuss weight without putting thinness on a pedestal and that obesity discourse “has eluded one of the greatest political, social, and cultural movements of the twentieth (and late nineteenth) century–feminism” (p. 250-251). We assert that social justice must be placed at the forefront of the discussion of weight bias, rather than as an indirect or subtle recognition. We echo Young's [70] statement that “there is no authentic, credible space where the oppression associated with fat, can be spoken about” (p. 251) and call for such credible spaces to be carved out in weight bias literature. It is in the direction of naming weight bias as a social justice issue that we invite continued debate between disciplines and an effort to find common ground to address the societal and structural inequalities that impact people with large bodies.

## 5. Conclusion

Although each of the different perspectives taken among weight bias researchers has unique approaches to understanding and examining weight bias; each perspective recognizes weight bias as an important social issue surrounding a discourse of weight as being within individual control. Each perspective has also identified specific social and individual consequences of weight bias, as well as the ways in which weight bias is related to social inequities. Our review of the common perspectives taken in weight bias research revealed differences in how weight bias is termed, conceptualized, and understood. However, it also revealed areas of convergence. Most notably, there is consensus among each perspective that weight bias is influenced by the pervasive belief that weight is within individual control. There is also consensus that weight bias has negative consequences ranging from individual health consequences to social oppression and that these consequences influence social inequity when intersected with gender, race, SES, and sexual orientation. Perhaps the consideration of such similarities, increased attention to social and structural inequalities, and openness to differing perspectives on language, including the use of both person-first and identity-first language, can fuel the future of research in weight bias to occur in a space where divergent views are respected in the name of social justice.

Although limited, previous researchers have also called for increased interdisciplinarity with regard to weight bias research and activism [69, 79]. Mansfield and Rich [79] stated that obesity discourse can be critically examined from multiple perspectives, but that those who critique discourse “are united by a common commitment and desire towards challenging dominant approaches to health which are solely weight-centric” (p. 359-360). They propose that researchers, practitioners, and policy makers who critique obesity discourse need to work across artificial barriers. They argue that increased involvement with the knowledge and perspectives of diverse fields may benefit weight bias activism [79]. Clare and colleagues [69] proposed that such integration may be fostered through applied interdisciplinarity, which focuses on strategically utilizing the knowledge and skills of various areas regarded as stakeholders of a specific issue.

In this discussion, we have invited researchers to recognize weight bias as an important social justice issue and to consider ways that our unique and combined efforts might address the aversive conditions under which body size is demarcated in our society as a space for the maltreatment and oppression of people. In addition, we called for researchers from various areas to work across professional boundaries in a joined effort for social change. Working towards increased interdisciplinarity between the various research areas and increased recognition of weight bias as an important social justice issue will serve people of every size.

## Figures and Tables

**Table 1:** Summary of similarities and differences identified among research areas.

Research perspective	Language used	Theoretical position	Consequences identified	Weight-based social inequity
Weight-centric	Obesity Obese person Person with obesity	Attribution theory Thin-ideal internalization Social comparison theory	Depression Anxiety Stress Binge-eating Low physical activity motivation Patient-provider relationship	Gender Patient quality of care

Health-centric	Fat/fatness Fat person	Critical analysis of obesity discourses Critical fat studies Feminist poststructuralism Intersectionality	Oppressive social context Harassment Social exclusion Female body anxiety Draining of female energy	Gender Race SES Sexual orientation

Health at every size	Obese/fat Fat person Obese person	Critical analysis of “healthism”	Reinforcing & privileging thinness Decontextualize health Demotivates health behaviour Avoidance of health care	
